# Mosaicism for GNAS methylation defects associated with pseudohypoparathyroidism type 1B arose in early post-zygotic phases

**DOI:** 10.1186/s13148-018-0449-4

**Published:** 2018-02-06

**Authors:** Francesca Marta Elli, Paolo Bordogna, Maura Arosio, Anna Spada, Giovanna Mantovani

**Affiliations:** 1 Endocrinology Unit, Department of Clinical Sciences and Community Health, University of Milan, Via Francesco Sforza, 35-20122 Milan, Italy; 2 Endocrinology and Metabolic Diseases Unit, Fondazione IRCCS Ca’ Granda Ospedale Maggiore Policlinico, Milan, Italy

**Keywords:** GNAS, Imprinting, Mosaicism, Pseudohypoparathyroidism, Albright hereditary osteodystrophy, PTH resistance, Epigenetics, Methylation defects

## Abstract

**Background:**

Pseudohypoparathyroidism type 1B (PHP1B; MIM#603233) is a rare imprinting disorder (ID), associated with the GNAS locus, characterized by parathyroid hormone (PTH) resistance in the absence of other endocrine or physical abnormalities. Sporadic PHP1B cases, with no known underlying primary genetic lesions, could represent true stochastic errors in early embryonic maintenance of methylation. Previous data confirmed the existence of different degrees of methylation defects associated with PHP1B and suggested the presence of mosaicism, a phenomenon already described in the context of other IDs.

**Results:**

With respect to mosaic conditions, the study of multiple tissues is a necessary approach; thus, we investigated somatic cell lines (peripheral blood and buccal epithelium and cells from the urine sediment) descending from different germ layers from 19 PHP patients (11 spor-PHP1B, 4 GNAS mutated PHP1A, and 4 PHP with no GNAS (epi)genetic defects) and 5 healthy controls. We identified 11 patients with epigenetic defects, further subdivided in groups with complete or partial methylation defects. The recurrence of specific patterns of partial methylation defects limited to specific CpGs was confirmed by checking methylation profiles of spor-PHP1B patients diagnosed in our lab (*n* = 56). Underlying primary genetic defects, such as uniparental disomy or deletion, potentially causative for the detected partial methylation were excluded in all samples.

**Conclusions:**

Our data showed no differences of methylation levels between organs and tissues from the same patient, so we concluded that the epimutation occurred in early post-zygotic phases and that the partial defects were mosaics. The number of patients with no detectable (epi)genetic GNAS defects was too small to exclude epimutations occurring in later post-zygotic phases, affecting only selected tissues different from blood, thus leading to underdiagnosis during routine molecular diagnosis. Finally, we found no correlation between methylation ratios, representing the proportion of epimutated cells, and the clinical presentation, further confirming the hypothesis of a threshold effect of the GNAS loss of imprinting leading to an “all-or-none” phenotype.

**Electronic supplementary material:**

The online version of this article (10.1186/s13148-018-0449-4) contains supplementary material, which is available to authorized users.

## Background

Pseudohypoparathyroidism type 1B (PHP1B; MIM # 603233) is a rare imprinting disorder (ID) characterized by renal resistance to parathyroid hormone (PTH) in the absence of other endocrine or physical abnormalities. Renal resistance to PTH, the hallmark of PHP, was documented by markedly blunted or absent urinary phosphate and cAMP responses to PTH injection. More recently, resistance to the action of thyroid-stimulating hormone (TSH) has also been described in a large subset of patients, while growth hormone (GH) secretion appears to be conserved [1–3].

In 2000, the PHP1B phenotype was associated with a loss of imprinting (LOI), at one or more GNAS differentially methylated regions (DMRs), which disrupts the parental-specific expression of its transcripts (the isolated, at the A/B DMR or the broad, at the NESP DMR associated with the simultaneous LOM at AS, XL and A/B DMRs) [2]. The autosomal dominant maternally transmitted form of the disorder (AD-PHP1B) is caused by microdeletions of long-range imprinting control elements (ICRs) that regulate GNAS imprinting [4–9]. Conversely, sporadic PHP1B cases (spor-PHP1B) present a broad GNAS LOI with no known underlying genetic lesion; thus, they could represent true stochastic errors in early embryonic maintenance of methylation [10]. In a small subset of PHP1B patients, the broad GNAS LOI resulted to be secondary to a complete or segmental uniparental disomy (UPD) of chromosome 20 [11, 12].

The DNA methylation undergoes a process of erasure, acquisition, and maintenance during gametogenesis, then it is maintained during the epigenetic reprogramming in the pre-implantation embryo, and disruptions in any of these steps may lead to IDs, resulting in the aberrant development of embryogenesis, placentation, and postnatal growth [13–15].

The examination of DNA methylation patterns in embryos and in corresponding sperm samples demonstrated no methylation errors at tested imprinted loci in sperm, suggesting that the paternal transmission of epigenetic abnormalities was unlikely [16]. The protection of imprint marks should mainly rely on maternal proteins stored in the oocyte before fertilization (i.e., *DPPA3*, *ZFP57*, *TRIM28*, and *DNMT1* gene products), because both the oocyte and the embryo are transcriptionally silent until the zygotic gene activation (ZGA), which takes place at the late four-cell stage in humans. This hypothesis was further supported by experiments in mouse embryos showing that ovarian stimulation disrupted maternal-effect gene products required for imprint maintenance [17].

The phenomenon of epigenetic mosaicism has been already described in the context of other IDs, such as Angelman syndrome (AS), Silver–Russell syndrome (SRS), Beckwith–Wiedemann syndrome (BWS), and transient neonatal diabetes (TND), and it was proposed that the absence of maternal gene products could cause complex chimeras with variable degrees of normally and aberrantly imprinted cells. Relaxation of IGF2 imprinting was previously found in BWS and in children with non-syndromic somatic overgrowth. In particular, the extent of and the location of (epi)genetic mosaicism may be responsible for the phenotype heterogeneity. Additionally, in most cases of TND, the hypomethylation was mosaic and more marked in non-leukocyte than in leukocyte samples [18–20].

The description of rare cases of monozygotic twins discordant for the methylation status of specific genes, which represent examples of post-zygotic epimutations arose after the twinning event, confirmed the existence of somatic mosaicism also for epigenetic defects [21, 22]. Single-cell DNA methylation analysis revealed the presence of epigenetic chimerism in pre-implantation embryos, emerging during the early pre-implantation phase of development, which translated into incomplete methylation patterns in whole embryos. However, further studies are needed to conclusively determine the mechanisms through which such chimerism is established [23].

Previously published data confirmed the existence of different degrees of methylation defects associated with PHP1B and suggested the presence of somatic mosaicism, with the contribution in a given tissue of both normal cells and cells with epigenetic defects [10, 24, 25]. Up to now, investigations were conducted on PHP patients’ DNA samples obtained from a single tissue, mainly peripheral blood, and no specific study has been performed to confirm and characterize the mosaicism associated with partial GNAS imprinting defects.

With respect to mosaic conditions, the study of multiple tissues is a necessary approach, so we collected the genetic material of somatic cell lines descending from different germ layers (ectoderm, mesoderm, and endoderm) that eventually gives rise to tissues and organs through the process of organogenesis, and we extensively studied their genetic and epigenetic status at the GNAS locus in both PHP patients and healthy controls.

## Results

We investigated genomic DNA samples obtained from different tissues of 5 healthy controls, 1 patient with PHP1A due to a large GNAS deletion, considered as a positive reference for methylation studies, and 19 PHP patients (11 spor-PHP1B, 4 GNAS-mutated PHP1A, and 4 PHP with no GNAS (epi)genetic defects).

Sanger sequencing analysis allowed to detect a point mutation in Gsα-coding exons in 4 patients (patients 12–15) and to exclude small genetic defects in the 15 other patients and 5 healthy controls. Methylation analysis identified 11 patients with broad GNAS LOI (raw methylation ratios in the Additional file 1: Table S1).

**Fig. 1:**
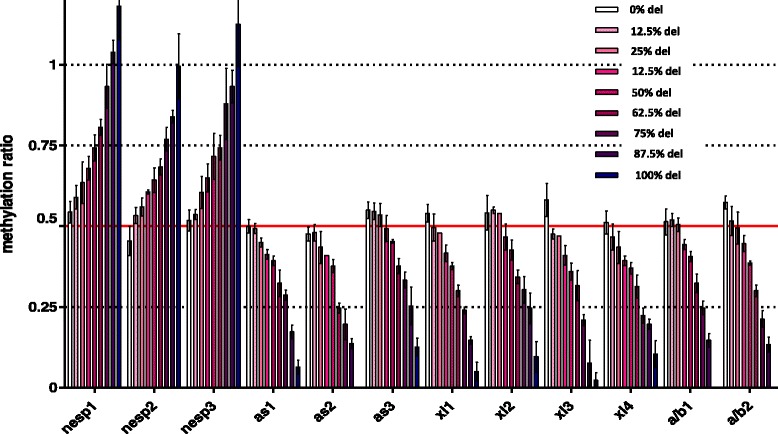
Graph summarizing methylation ratio ranges detected by MS-MLPA using a 9-point serial dilution of gDNA from a PHP patient affected by a deletion of the GNAS locus on the maternal allele, from 0% (wild type, hemimethylated gDNA) to 100% (severe LOI) GNAS-deleted DNA. On the *Y* axis, mean ± SD observed methylation ratios are reported, while on the *X* axis, the 12 analyzed CpGs representative of GNAS DMRs. The red line in the *Y* axis highlights the 0.5 methylation ratio value expected in wild-type samples

The comparison of raw methylation ratios confirmed the reproducibility and reliability of the methylation specific–multiplex ligand-dependent probe amplification (MS-MLPA) assay, allowing data validation for subsequent statistical analysis. Data obtained from the serial dilution of a sample with a GNAS maternal deletion confirmed the ability to detect different levels of methylation (Fig. 1).

After the analysis of genetic markers upstream and downstream the GNAS locus to determine whether UPD could be the primary genetic cause of the LOI, all cases resulted having at least one heterozygous VNTR in the 20q region (Additional file 1: Table S2). In patients 2 and 11, we were also able to exclude uniparental heterodisomy as parent’s DNA was available. The exclusion of uniparental isodisomy and aneuploidies as causative genetic aberration further supported that the detection of partial LOI is derived from the presence of an epigenetic mosaicism rather than a genetic mosaicism.

**Fig. 2:**
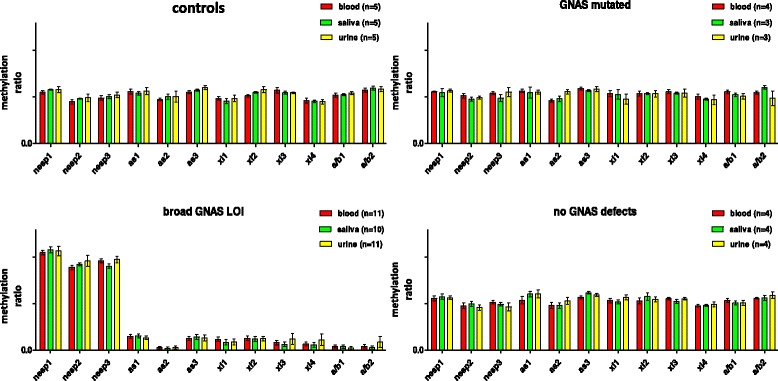
Graphs showing methylation ratios (mean blood/saliva/urine replicates ± SD) at each single CpG for investigated subjects grouped as wild type controls (WT ctrls), patients bearing genetic point mutations at the *GNAS* gene (GNAS mutated), patients affected by methylation defects at all four GNAS DMRs (broad GNAS LOI), and patients with a clinical diagnosis of PHP but no detectable genetic and/or epigenetic GNAS defects (no GNAS defects)

Single CpG methylation ratios in the three tissues (peripheral blood and buccal epithelium and cells from the urine sediment) were the same for each subject, and in the subsequent statistical analysis, we considered mean values (Table 1 and Fig. 2).

**Table 1:** Table resuming observed by MS-MLPA methylation ratio means (± SD) at GNAS CpGs (three for NESP DMR, three for AS DMR, four for XL DMR, and two for A/B DMR)

		NESP1		NESP2		NESP3		AS1		AS2		AS3		XL1		XL2		XL3		XL4		A/B1		A/B2	
Sample	Tissue	Mean	SD	Mean	SD	Mean	SD	Mean	SD	Mean	SD	Mean	SD	Mean	SD	Mean	SD	Mean	SD	Mean	SD	Mean	SD	Mean	SD
Ctrls	B (5)	0.55	0.05	0.45	0.05	0.49	0.05	0.56	0.06	0.47	0.03	0.55	0.04	0.48	0.04	0.51	0.03	0.57	0.07	0.46	0.06	0.52	0.05	0.57	0.05
Ctrls	S (5)	0.58	0.01	0.48	0.02	0.50	0.04	0.54	0.04	0.50	0.06	0.57	0.02	0.46	0.06	0.55	0.02	0.55	0.04	0.45	0.03	0.52	0.02	0.60	0.05
Ctrls	U (5)	0.58	0.07	0.49	0.09	0.52	0.06	0.56	0.09	0.50	0.13	0.60	0.05	0.48	0.08	0.58	0.08	0.54	0.02	0.45	0.06	0.54	0.04	0.58	0.06
Mut pts	B (4)	0.56	0.02	0.51	0.05	0.54	0.03	0.57	0.04	0.46	0.03	0.59	0.03	0.54	0.06	0.54	0.06	0.56	0.04	0.50	0.05	0.56	0.04	0.55	0.03
Mut pts	S (3)	0.55	0.08	0.48	0.04	0.49	0.06	0.55	0.11	0.48	0.05	0.57	0.02	0.53	0.09	0.54	0.02	0.54	0.02	0.48	0.02	0.52	0.04	0.60	0.03
Mut pts	U (3)	0.57	0.03	0.49	0.03	0.55	0.09	0.55	0.04	0.56	0.04	0.59	0.05	0.48	0.09	0.53	0.06	0.54	0.07	0.47	0.09	0.51	0.05	0.49	0.14
No def pts	B (4)	0.56	0.06	0.48	0.06	0.52	0.04	0.54	0.08	0.48	0.06	0.57	0.03	0.54	0.05	0.53	0.07	0.56	0.03	0.48	0.03	0.54	0.04	0.56	0.01
No def pts	S (4)	0.57	0.06	0.49	0.05	0.49	0.06	0.61	0.06	0.45	0.09	0.61	0.02	0.51	0.06	0.57	0.07	0.51	0.06	0.46	0.05	0.51	0.05	0.55	0.08
No def pts	U (4)	0.58	0.05	0.47	0.04	0.46	0.09	0.60	0.08	0.55	0.1	0.61	0.03	0.56	0.04	0.54	0.06	0.55	0.04	0.50	0.07	0.51	0.06	0.59	0.08
Del pt	B (1)	1.05	0.08	0.90	0.02	1.04	0.15	0.00	0.00	0.00	0.00	0.05	0.07	0.00	0.00	0.08	0.01	0.00	0.00	0.00	0.00	0.00	0.00	0.00	0.00
PHP1B pts	B (11)	1.05	0.13	0.89	0.11	0.96	0.12	0.15	0.07	0.03	0.06	0.13	0.08	0.12	0.08	0.13	0.08	0.09	0.09	0.07	0.08	0.05	0.06	0.04	0.06
PHP1B pts	S (10)	1.08	0.16	0.92	0.13	0.90	0.12	0.15	0.07	0.02	0.05	0.14	0.09	0.08	0.09	0.12	0.09	0.06	0.08	0.05	0.08	0.04	0.07	0.02	0.06
PHP1B pts	U (11)	1.08	0.19	0.97	0.22	0.98	0.16	0.13	0.07	0.03	0.06	0.12	0.11	0.07	0.10	0.12	0.09	0.09	0.16	0.08	0.16	0.02	0.05	0.06	0.16

Data in parenthesis are the number of samples reported

*B* blood, *S* saliva, *U* urine

As expected, in healthy samples and in PHP patients affected by GNAS point mutations, the NESP DMR was hemimethylated on the paternal allele, while AS, XL, and A/B DMRs on the maternal allele.

The investigation of different tissues of four patients with no GNAS (epi)genetic defects failed to find an imprinting defect in cell lineages different from leucocytes, which could explain the clinical phenotype in the absence of anomalies in blood samples.

**Fig. 3:**
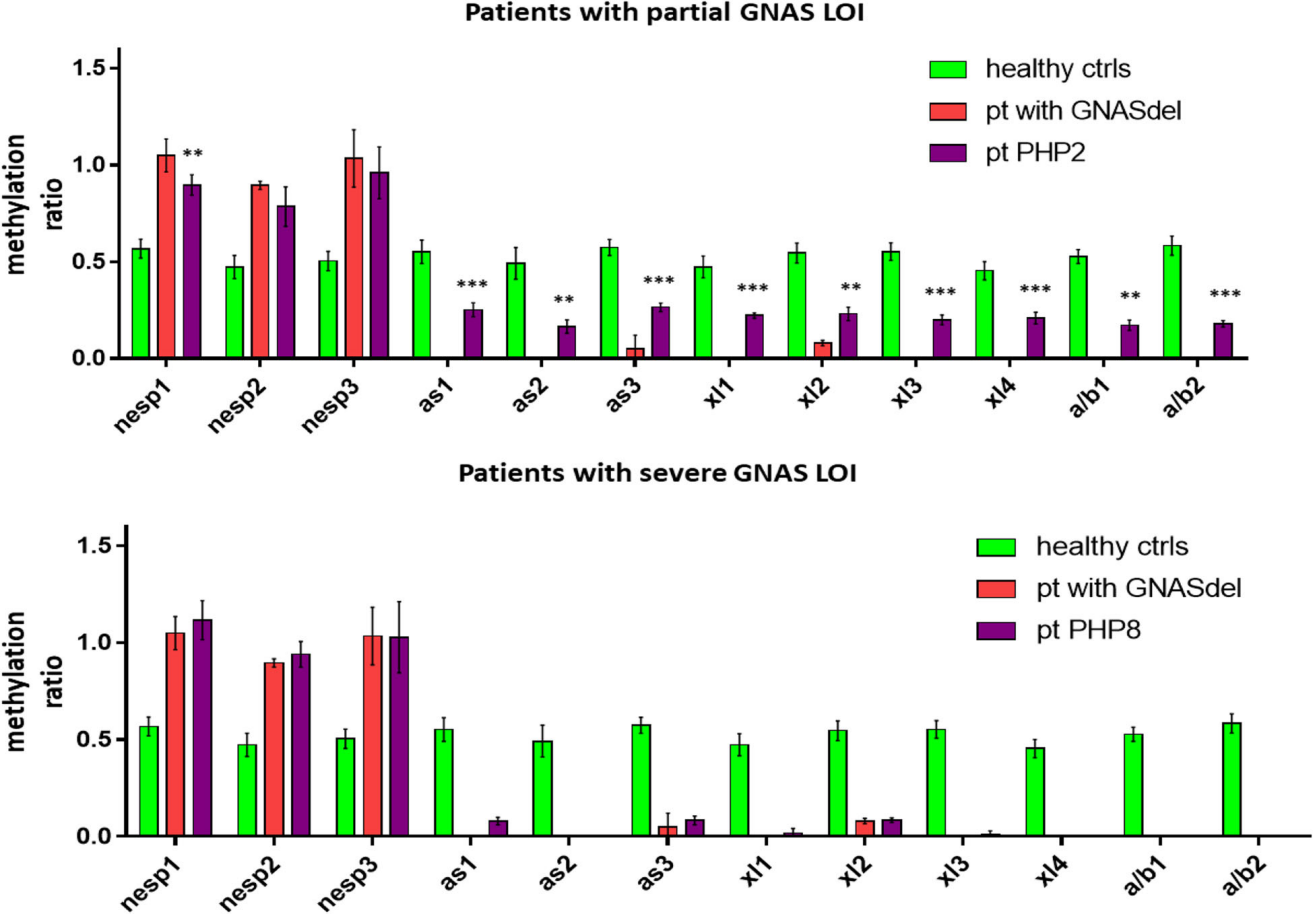
Graphs showing the methylation ratio (mean blood/saliva/urine replicates ± SD) at each investigated CpG for two representative spor-PHP1B patients with partial (PHP2) and severe (PHP8) loss of imprinting (LOI) at GNAS DMRs compared to values obtained in healthy controls (negative ctrls) and the patient with the GNAS locus deletion (positive ctrl). All methylation ratios observed in spor-PHP1B patients were significantly different from those in wild type (*P* < 0.001). Methylation ratios determined in spor-PHP1B patients could be not significantly different (no symbol above the column) or significantly different (*P < 0.05; **P < 0.01; ***P < 0.001) from those due to ablation of the maternal allele

Because of the variability among different CpG methylation ratios belonging to the same DMR in spor-PHP1B with respect to negative and positive references (NESP DMR = 0.97 ± 0.17 (mean of three CpGs), AS DMR = 0.11 ± 0.11 (mean of three CpGs), XL DMR = 0.10 ± 0.11 (mean of four CpGs), and A/B DMR = 0.05 ± 0.10 (mean of two CpGs)), we considered single CpGs instead of whole DMRs in the downstream analysis. This CpG-specific analysis (raw data in the Additional file 1: Table S1) allowed subdivision of spor-PHP1B patients in two major groups, one with a “complete” methylation defect (*n* = 6) and another with a “partial” methylation defect (*n* = 5) (Fig. 3).

The “partial” cluster could be further subdivided for the presence of a partial methylation defect limited to specific CpGs. Only one patient (patient 2) showed a partial methylation defect in all CpGs, with methylation ratios significantly different (*P* values < 0.01 or < 0.001) from the positive deleted control. Conversely, the other four patients displayed a partial LOM limited to the AS DMR, the XL DMR, or both the AS and the XL DMRs. In particular, patient 5 had a partial LOM at the AS DMR (AS1 = 0.24 ± 0.03 and AS3 = 0.25 ± 0.04) and patient 1, a partial LOM at the XL DMR (XL1 = 0.22 ± 0.05, XL2 = 0.25 ± 0.04, XL3 = 0.18 ± 0.11, and XL4 = 0.17 ± 0.01), while patients 3 and 4 had a partial LOM at both AS and XL DMRs (AS1 = 0.21 ± 0.04/0.17 ± 0.02, AS3 = 0.21 ± 0.06/0.15 ± 0, XL1 = 0.20 ± 0.03/0.17 ± 0.01, XL2 = 0.22 ± 0.03/0.13 ± 0.01, XL3 = 0.27 ± 0.22/0.13 ± 0.02, and XL4 = 0.24 ± 0.24/0.09 ± 0, respectively) (Additional file 1: Table S1).

The methylation status of the A/B DMR of partial patients, with the exception of patient PHP2, resulted more severely affected by the LOM when compared with AS and XL DMRs, with the exclusion of the AS CpG number 2 whose methylation ratio was nearly 0 in all samples. This observation further supported the hypothesis of the existence of a second independent ICR regulating their epigenetic status, independently from the A/B DMR. Moreover, the gain of methylation (GOM) at the NESP DMR was complete in all partial samples, except in patient PHP2.

**Fig. 4:**
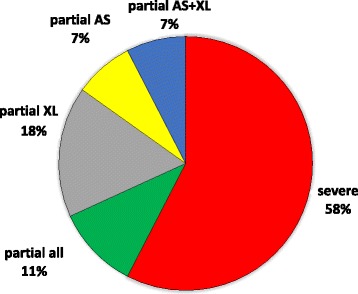
Pie chart showing the prevalence of different sub-clusters of methylation defects detected in our cohort of PHP1B patients with broad GNAS imprinting defects. In particular, patients were firstly divided into two main groups, the severe and the partial methylation defect (58 and 42%, respectively), and then the partial group was further subdivided into partial LOM limited to the XL DMR (18%, 12 of 67 pts), partial LOM limited to the AS DMR (7%, 5 of 67 pts), partial LOM limited to AS and XL DMRs (7%, 5 of 67 pts), and partial LOI at all 4 DMRs (10%, 7 of 67 pts)

To confirm our findings about “partial LOM” sub-clusters, we checked GNAS methylation profiles obtained from genomic DNA extracted from peripheral blood samples in our series of spor-PHP1B patients diagnosed in our lab (*n* = 56), and we observed that these patterns of partial LOM were recurrent. In particular, if we consider the whole cohort of spor-PHP1B patients (*n* = 67), we found that the prevalence of cases with partial epigenetic anomalies was about 42%: 5 patients had a partial LOM limited to the AS DMR, 12 patients had a partial LOM limited to the XL DMR, 5 patients had partial LOMs at AS and XL DMRs, and 7 patients had partial methylation defects at all 4 DMRs (Fig. 4).

Finally, we searched for an explanation of the phenotypic variability, which means the number or severity of hormonal resistances and the presence of signs of Albright hereditary osteodystrophy (AHO), according to the methylation ratio and/or the specific affected CpGs, but we failed to find any significant difference among patients with or without AHO rather than with one or more endocrine alterations.

## Discussion

In the present work, we investigated GNAS imprinting in DNA samples obtained from different tissues of a small series of PHP patients, as previously published data confirmed the existence of different degrees of methylation defects associated with PHP1B and suggested the presence of epigenetic mosaicism [10, 19].

The broad GNAS LOI was previously associated with complete or segmental 20qUPD in small subsets of patients, but this hypothesis was unlikely in our case series as all patients were heterozygous for at least one genetic marker, further supporting the presence of epigenetic mosaicism in subjects with partial methylation defects [11, 12].

To study mosaic conditions, the experimental approach relies on the analysis of multiple tissues from the same subject; thus, we collected the genetic material of cell lines descending from different germ layers (ectoderm, mesoderm, and endoderm). We did not observe significant differences in methylation levels in different tissues from the same patient, so we concluded that the partial LOI was a mosaic condition due to an epimutation which occurred in early post-zygotic phases, before the germ layers’ formation. In particular, in patients with partial LOI, we determined methylation ratios of 0.15–0.25, consistent with an error in methylation that occurred at the four- or eight-cell phase, i.e., the phase during which the post-zygotic genome-wide passive demethylation takes place [13–17].

Following fertilization, parental genomes undergo a global demethylation to facilitate the remodeling from two distinct differentiated gamete-specific states to a pluripotent embryonic state, with the exception of imprinted loci whose differential methylation is preserved by maternal zygotic effect genes accumulated during the oocyte growth. Maternal-effect proteins that protect imprinted methylation sites during pre-implantation development identified up to now include developmental pluripotency-associated 3 (DPPA3; also called STELLA/PGC7), zinc finger protein 57 (ZFP57), tripartite motif-containing 28 protein (TRIM28; also called KAP1/TIF1b) and DNA methyltransferase 1 (DNMT1) [16, 17]. Mutations in ZFP57 were associated with TND, the first heritable global ID compatible with life described in humans, and perhaps additional methylation anomalies of imprinted loci are not yet described [26].

Although the underlying molecular cause of spor-PHP1B is not clear, the mosaicism in cases with partial GNAS methylation defects is consistent with a failure of methylation maintenance in the early zygote, and a maternal-effect mutation might be postulated. On the other hand, in patients affected by complete broad methylation defects, an imprinting defect originating in gametes, resulting in unstable methylation of the ICRs afterwards, could be considered, but further studies to detect factors associated with the GNAS imprinting maintenance are needed. Conversely, defects affecting cells from a specific tissue would derive from molecular errors during later phases of the embryo development, but unfortunately, the number of investigated patients with no detectable GNAS (epi)genetic defects was too small to fully exclude epimutations occurring in later post-zygotic phases.

Studies in the mouse model performed to investigate structure and regulation of the GNAS-imprinted cluster identified 2 candidate ICRs with a different mode of action, one at exon A/B, acting as an insulator model, and one covering AS and XL promoters, acting through a mechanism involving antisense RNAs [27]. The detection of different patterns of mild epigenetic anomalies in our patients, in particular, partial LOMs limited to the AS DMR, the XL DMR, or both AS and XL DMRs associated with a complete LOM at the A/B DMR, uphold the assumption that two independent and interacting ICRs regulate GNAS methylation and highlighted the hypothesis that different molecular mechanisms might be involved in the epigenetic dysregulation associated to PHP1B. Additionally, a recent work published by Court and colleagues showed that the ICR in the AS-XL region is not a single regulatory unit and that its two DMRs are partitioned by an interval of about 200-bp [28]. These data were in accordance with our finding of patients with partial LOM limited to the AS DMR or to the XL DMR.

Some DMRs, defined as germline or primary DMRs, acquire their allelic methylation during gametogenesis and are stably maintained throughout the post-zygotic development. Conversely, somatic or secondary DMRs acquire methylation during development and are regulated by nearby germline DMRs [28]. Previous studies found that AS, XL, and A/B DMRs are germline DMRs and that the AS DMR affects the somatic NESP DMR methylation status, as its antisense transcript could act in a similar manner to Air [29]. As a matter of fact, the isolated LOM at the A/B DMR, both in sporadic and inherited patients, does not affect the imprinting of the NESP DMR [30]. Our observations further supported this hypothesis because the GOM at the NESP DMR was complete also in partial patients, except for patient 2, who displayed a complete LOM at the CpG AS2, raising the possibility that this region contains a *cis*-acting sequence involved in the NESP DMR methylation establishment.

Finally, in our previous work, conducted in 63 patients with PHP type 1 and GNAS imprinting defects, we found no correlation between the degree of methylation defects and the severity of clinical parameters [25]. The absence of correlation between methylation ratios and the phenotype observed in the present study further confirms our previous findings and can be explained by a threshold effect leading to an “all-or-none” phenotype.

## Conclusions

To conclude, further studies to unravel the mechanisms underlying GNAS LOI are needed and, in particular, it will be crucial to determine whether GNAS LOI in spor-PHP1B patients is associated with a primary or secondary epigenetic defect, with the final aim to correctly evaluate the recurrence risk of this imprinting syndrome.

## Methods

The present study included 5 healthy controls, 1 subject affected by a whole GNAS locus deletion (g.56′657′752_59′228′953del, previously described in reference 34), used as positive reference for methylation defects at GNAS DMRs, and 19 PHP patients (11 with methylation defects at the GNAS locus, 4 with point mutations in GNAS Gsα-coding exons, and 4 with no GNAS/PDE4D/PRKAR1A (epi)genetic defects and no deletions of the long arm of chromosome 2). Some of these PHP patients (1–6, 9–10, 12–14, and 16–17) were presented in our previous works [25, 28, 29, 31]. The clinical diagnosis was based upon the presence or absence of specific clinical and biochemical signs, i.e., hypocalcemia, hyperphosphatemia, and raised serum PTH levels in the absence of vitamin D deficiency and/or typical AHO manifestations, considered major and minor criteria for the diagnosis of PHP [32–35]. Clinical details, including some additional patient-specific features, and molecular diagnosis are summarized in Table 2. Informed consent for genetic and epigenetic studies was obtained from all subjects involved in the study.

**Table 2:** Clinical characteristics and molecular analysis of patients included in the present study

pt ID	GNAS point mut	GNAS meth def	Sex	PTH	TSH	SS	Ob	RF	Br	MR/BD	OS	Additional features
1	No	Yes	F	*183*	2.7							
2	No	Yes	M	*899*	2.6		Yes					Long QT syndrome
3	No	Yes	M	*258*	*4.1*							Hypertension, Fahr syndrome, and renal damage
4	No	Yes	F	*215*	2.5							
5	No	Yes	M	*454*	*4.9*							
6	No	Yes	M	*114*	*5.4*							Subclinic hypothyroidism
7	No	Yes	M	*X*					Yes			Long QT syndrome
8	No	Yes	F	*152*	0.29					Yes		Leukopenia, normocytic microcromica anemia, autoimmune primary hypothyroidism, and osteopenia
9	No	Yes	M	*288*	*7*							Asperger syndrome
10	No	Yes	F	*615*	1.8							Asthenia associated with lipothymia, genu valgo, andhypertension
11	No	Yes	M	*338*	2.1		Yes					LGA at birth
12	Yes	No	F	*373*	*8.34*						Yes	Popliteal cyst, aortic stenosis, rGHRH, and genu valgo
13	Yes	No	F	*83.6*	1.49	Yes			Yes		Yes	Irregular menses and hypertension
14	Yes	No	F	*X*	*4.48*							
15	Yes	No	F	*138*	*34*					Yes		Oligohydramnios,congenital hyperthyroidism, rickets, spastic diplegia, and amenorrhea
16	No	No	F	10.6	*7.9*		Yes	Yes	Yes	Yes		Cholelithiasis, hypoadrenalism
17	No	No	M	*77*	*X*				Yes			Congenital adrenal hyperplasia
18	No	No	F	*77.3*	1.65							Hashimoto thyroiditis, favism, and multiple fractures
19	No	No	F	37	*X*		Yes					Oligomenorrhoea

Age (years at diagnosis)

*PTH* PTH serum levels, normal range 10–65 pg/mL, *TSH* TSH serum levels, normal range 0.4–3.9 mU/L, *X* elevated, but value not available, *Ob* obesity, *RF* round face, *MR/BD* mental retardation and/or behavioral defects, *Br* brachydactyly, *OS* ectopic ossifications, *SS* short stature, *LGA* large for gestational age

Genomic DNA was extracted from samples of the peripheral blood (Nucleon BACC2 genomic DNA purification kit, cod.RPN8502, GE Healthcare), saliva, and urine (Puregene Core kit, cod. 158622, Qiagen), in order to obtain the genetic material from somatic cell lines descending from different germ layers. Buccal epithelial cells are of ectodermal derivation and urine sediment cells are derived from the mesoderm, while leukocytes are from the endoderm. Mutation analysis was performed by direct sequencing (BigDye™Terminator v3.1 Cycle Sequencing Kit, cod.4337456, Applied Biosystems) of GNAS 1–13 exons and flanking intronic sequences (ENSEMBL ID: ENSG00000087460). Informative genetic markers in the 20q region were evaluated to exclude a possible misdiagnosis of spor-PHP1B in the presence of aneuploidies (monosomy or trisomy) or uniparental disomy (UPD). According to sample availability, the analysis was performed in blood, saliva, and urine samples. For variable number tandem repeat (VNTR) genotyping, we set up a cost-effective three-primer approach based on the simultaneous use of a couple of sequence-specific primers, the reverse one containing a poliA tail at the 5′ end to allow easier allele scoring, associated with a fluorescently labeled universal forward M13-tailed FAM oligonucleotide. All primers and experimental conditions are available upon request. Copy number variant (CNV) analysis of STX16 and GNAS loci and methylation analysis of GNAS DMRs were performed by MS-MLPA (cod. ME031-100R+ EK1-FAM, MRC-Holland) (methylation-specific probe location, GR37/hg19, summarized in Additional file 1: Figure S1). MS-MLPA data analysis was performed using the Coffalyser.Net (MRC-Holland). The detection of expected MS-MLPA methylation ratios of about 0.5 reflected a normal hemimethylated status in healthy controls (considered as a negative reference), while finding of MS-MLPA methylation ratios of about 1 and 0 reflected the apparent LOI in the GNAS-locus-deleted subject (considered as positive reference). At least two technical replicates were performed for each biological sample. A serial dilution, in triplicate, of GNAS-deleted DNA (0, 12.5, 25, 37.5, 50, 62.5, 75, 87.5, and 100%) in wild type DNA was created to define the MS-MLPA detection range of different percentages of epigenetic mosaics. Statistical analysis was performed using GraphPad Prism version 5 for Windows (www.graphpad.com). The exploratory data analysis included means ± SD, median, and interquartile ranges for values measured in the blood, saliva, and urine. Based on data distribution, means ± SD were used for further comparisons. One-way ANOVA with Bonferroni’s Multiple Comparison test was used to evaluate the intercluster variability at single CpGs. After Bonferroni correction, a *P* value < 0.05 was deemed to indicate statistical significance. Continuous variables, i.e., hormonal and biochemical parameters, were reported as means ± SD, whereas dichotomous variables, i.e., the presence of AHO signs, were expressed as proportions. Differences between means and proportions were checked by Student’s *t* and chi-squared tests, respectively. In particular, for the epigenotype–phenotype correlation investigation, we considered the following clinical parameters: the precocity of the disease (defined as the age at diagnosis), the severity of hormonal resistances (defined as PTH, calcium, phosphorus, and TSH levels at diagnosis), and the severity of AHO, defined as the number of AHO signs.

## Additional file

Additional file 1: Figure S1. Methylation-specific probes location, GR37/hg19. **Table S1**. Raw methylation ratios. **Table S2**. Variable number tandem repeats (VNTRs) in the 20q region analysed to exclude uniparental disomy (UPD). (DOCX 378 kb)

## Abbreviations

AHO: Albright hereditary osteodystrophy
AS: Angelman syndrome
BWS: Beckwith–Wiedemann syndrome
cAMP: Cyclic adenosine monophosphate
DMRs: Differentially methylated regions
GH: Growth hormone
GOM: Gain of methylation
Gsα: Alpha subunit of the stimulatory G protein
ICRs: Imprinting control elements
ID: Imprinting disorder
LOI: Loss of imprinting
LOM: Loss of methylation
MS-MLPA: Methylation-specific–multiplex ligand-dependent probe amplification
PHP: Pseudohypoparathyroidism
PTH: Parathyroid hormone
SD: Standard deviation
SRS: Silver–Russell syndrome
TND: Transient neonatal diabetes
TSH: Thyroid-stimulating hormone
UPD: Uniparental disomy
VNTR: Variable number tandem repeat
ZGA: Zygotic gene activation
