# Supplementary material for: Mosaicism for GNAS methylation defects associated with pseudohypoparathyroidism type 1B arose in early post-zygotic phases
Source: Clin Epigenetics. 2018 Feb 6;10:16. doi: 10.1186/s13148-018-0449-4 (PMC5801752; doi:10.1186/s13148-018-0449-4)

**Supplementary Table 1**

| **pt ID** |  | **nesp1** | **nesp2** | **nesp3** | **as1** | **as2** | **as3** | **xl1** | **xl2** | **xl3** | **xl4** | **ab1** | **ab2** |
| --- | --- | --- | --- | --- | --- | --- | --- | --- | --- | --- | --- | --- | --- |
| **1** | **B1** | 1.06 | 0.78 | 0.93 | 0.11 | 0 | 0.11 | 0.19 | 0.23 | 0.14 | 0.16 | 0 | 0 |
|  | **B2** | 1.08 | 1.01 | 1.11 | 0.08 | 0 | 0.09 | 0.27 | 0.28 | 0.3 | 0.18 | 0 | 0 |
|  | **S1** | 1 | 0.67 | 0.75 | 0.1 | 0 | 0.14 | 0.17 | 0.24 | 0.18 | 0.19 | 0 | 0 |
|  | **S2** | 1.52 | 1.19 | 1.14 | 0.13 | 0 | 0.07 | 0.21 | 0.22 | 0 | 0.17 | 0 | 0 |
|  | **U1** | 1.01 | 0.93 | 1.28 | 0.07 | 0 | 0.12 | 0.21 | 0.23 | 0.21 | 0.16 | 0 | 0 |
|  | **U2** | 0.87 | 0.99 | 0.97 | 0.07 | 0 | 0.11 | 0.29 | 0.31 | 0.26 | 0.18 | 0 | 0 |
| **2** | **B1** | 0.88 | 0.72 | 1.06 | 0.28 | 0.16 | 0.27 | 0.24 | 0.22 | 0.18 | 0.21 | 0.17 | 0.17 |
|  | **B2** | 0.86 | 0.69 | 0.85 | 0.22 | 0.19 | 0.25 | 0.21 | 0.22 | 0.15 | 0.2 | 0.15 | 0.17 |
|  | **B3** | 0.89 | 0.75 | 0.82 | 0.3 | 0.13 | 0.26 | 0.2 | 0.21 | 0.21 | 0.16 | 0.17 | 0.16 |
|  | **S1** | 0.86 | 0.83 | 0.93 | 0.23 | 0.18 | 0.24 | 0.23 | 0.28 | 0.22 | 0.23 | 0.17 | 0.19 |
|  | **S2** | 0.99 | 0.94 | 0.86 | 0.29 | 0.16 | 0.27 | 0.23 | 0.24 | 0.22 | 0.19 | 0.22 | 0.18 |
|  | **U1** | 0.85 | 0.89 | 1.19 | 0.24 | 0.22 | 0.31 | 0.22 | 0.27 | 0.22 | 0.25 | 0.19 | 0.18 |
|  | **U2** | 0.95 | 0.68 | 1.01 | 0.21 | 0.12 | 0.26 | 0.23 | 0.18 | 0.2 | 0.23 | 0.14 | 0.21 |
| **3** | **B1** | 0.94 | 0.91 | 1.09 | 0.19 | 0 | 0.19 | 0.21 | 0.2 | 0.15 | 0.17 | 0.1 | 0.12 |
|  | **B2** | 1.06 | 0.81 | 1.09 | 0.27 | 0 | 0.12 | 0.16 | 0.27 | 0.2 | 0.12 | 0.06 | 0.11 |
|  | **S1** | 1.06 | 0.79 | 0.89 | 0.19 | 0 | 0.22 | 0.17 | 0.19 | 0.18 | 0.12 | 0.08 | 0 |
|  | **S2** | 1.14 | 1.07 | 0.99 | 0.24 | 0 | 0.26 | 0.23 | 0.24 | 0.16 | 0.13 | 0.14 | 0.11 |
|  | **U** | 0.9 | 0.83 | 0.96 | 0.18 | 0.01 | 0.28 | 0.21 | 0.2 | 0.67 | 0.67 | 0 | 0.67 |
| **4** | **B** | 1.08 | 0.81 | 1.04 | 0.18 | 0.05 | 0.15 | 0.16 | 0.13 | 0.11 | 0.09 | 0.06 | 0.08 |
|  | **S** | na | na | na | na | na | na | na | na | na | na | na | na |
|  | **U** | 0.94 | 0.79 | 0.84 | 0.15 | 0.08 | 0.15 | 0.17 | 0.12 | 0.14 | 0.09 | 0.04 | 0.05 |
| **5** | **B1** | 0.85 | 0.85 | 0.96 | 0.21 | 0 | 0.2 | 0.06 | 0.04 | 0 | 0 | 0 | 0 |
|  | **B2** | 1.19 | 0.91 | 0.76 | 0.24 | 0 | 0.23 | 0.06 | 0.1 | 0 | 0 | 0 | 0 |
|  | **S1** | 0.98 | 0.66 | 0.78 | 0.24 | 0 | 0.21 | 0.04 | 0.03 | 0.03 | 0 | 0 | 0 |
|  | **S2** | 1.26 | 0.96 | 0.7 | 0.3 | 0 | 0.3 | 0.1 | 0.09 | 0 | 0 | 0.04 | 0 |
|  | **U1** | 0.97 | 0.69 | 0.75 | 0.23 | 0 | 0.28 | 0 | 0.05 | 0 | 0 | 0 | 0 |
|  | **U2** | 0.98 | 0.81 | 0.8 | 0.22 | 0 | 0.26 | 0.05 | 0.08 | 0 | 0 | 0 | 0 |
| **6** | **B1** | 0.98 | 0.99 | 1.01 | 0.1 | 0.02 | 0.09 | 0.08 | 0.04 | 0.05 | 0 | 0.02 | 0 |
|  | **B2** | 1.12 | 1.01 | 0.88 | 0.1 | 0 | 0.1 | 0.06 | 0.09 | 0.05 | 0 | 0 | 0 |
|  | **S1** | 1.05 | 0.86 | 0.91 | 0.11 | 0 | 0.1 | 0.03 | 0.03 | 0.03 | 0 | 0 | 0 |
|  | **S2** | 1.13 | 1.03 | 0.9 | 0.08 | 0 | 0.05 | 0 | 0.08 | 0 | 0 | 0 | 0 |
|  | **U1** | 1.19 | 1.15 | 1.26 | 0.12 | 0.01 | 0 | 0.01 | 0 | 0.02 | 0.03 | 0.01 | 0.03 |
|  | **U2** | 1.2 | 1.03 | 0.92 | 0.1 | 0 | 0 | 0 | 0.08 | 0 | 0 | 0 | 0 |
| **7** | **B1** | 1 | 0.92 | 1.13 | 0.12 | 0 | 0.12 | 0.11 | 0.13 | 0.08 | 0 | 0.04 | 0 |
|  | **B2** | 1.23 | 1.03 | 0.83 | 0.16 | 0 | 0.15 | 0.12 | 0.12 | 0.09 | 0.07 | 0.09 | 0.06 |
|  | **S1** | 0.87 | 0.93 | 1.03 | 0.1 | 0 | 0.12 | 0 | 0.11 | 0.08 | 0 | 0 | 0 |
|  | **S2** | 1.1 | 0.93 | 0.78 | 0.16 | 0 | 0.15 | 0.08 | 0.13 | 0.07 | 0.05 | 0.06 | 0 |
|  | **U1** | 0.79 | 0.71 | 1.07 | 0.1 | 0 | 0.09 | 0 | 0.06 | 0.09 | 0 | 0 | 0 |
|  | **U2** | 1.19 | 1.03 | 0.86 | 0.17 | 0.08 | 0.17 | 0.09 | 0.16 | 0 | 0.05 | 0.07 | 0 |
| **8** | **B1** | 1.02 | 0.91 | 1.14 | 0.07 | 0 | 0.06 | 0 | 0.08 | 0.05 | 0 | 0 | 0 |
|  | **B2** | 1.26 | 1.04 | 0.87 | 0.1 | 0 | 0.1 | 0.05 | 0.1 | 0 | 0 | 0 | 0 |
|  | **S1** | 1.05 | 0.87 | 1.19 | 0.06 | 0 | 0.07 | 0 | 0.08 | 0 | 0 | 0 | 0 |
|  | **S2** | 1.08 | 0.98 | 0.86 | 0.09 | 0 | 0.1 | 0.05 | 0.1 | 0 | 0 | 0 | 0 |
|  | **U1** | 1.06 | 0.88 | 1.25 | 0.06 | 0 | 0.06 | 0 | 0.07 | 0 | 0 | 0 | 0 |
|  | **U2** | 1.23 | 0.96 | 0.86 | 0.1 | 0 | 0.11 | 0 | 0.08 | 0 | 0 | 0 | 0 |
| **9** | **B1** | 0.99 | 0.73 | 0.87 | 0.09 | 0 | 0.11 | 0.08 | 0.05 | 0 | 0 | 0.02 | 0 |
|  | **B2** | 1.29 | 1.05 | 0.87 | 0.08 | 0 | 0.09 | 0.05 | 0.09 | 0 | 0 | 0 | 0 |
|  | **S1** | 1.04 | 0.79 | 0.93 | 0.1 | 0 | 0.12 | 0 | 0.03 | 0.04 | 0 | 0 | 0 |
|  | **S2** | 1.32 | 1.04 | 0.86 | 0.1 | 0 | 0.09 | 0 | 0.1 | 0 | 0 | 0 | 0 |
|  | **U1** | 1.19 | 0.85 | 0.8 | 0.1 | 0.01 | 0.12 | 0 | 0.06 | 0 | 0 | 0 | 0.01 |
|  | **U2** | 1.43 | 1.08 | 0.95 | 0.14 | 0 | 0.11 | 0 | 0.12 | 0 | 0 | 0 | 0 |
| **10** | **B1** | 1.03 | 0.82 | 1.1 | 0.1 | 0.11 | 0.13 | 0.12 | 0.1 | 0.06 | 0.13 | 0.08 | 0.07 |
|  | **B2** | 1.21 | 0.94 | 0.84 | 0.11 | 0 | 0 | 0.12 | 0.09 | 0 | 0 | 0 | 0 |
|  | **S1** | 1.08 | 1 | 0.85 | 0.15 | 0 | 0.11 | 0.12 | 0.09 | 0 | 0 | 0 | 0 |
|  | **S2** | 0.89 | 1.01 | 0.82 | 0.14 | 0 | 0.14 | 0 | 0.1 | 0 | 0 | 0 | 0 |
|  | **U1** | 1.21 | 1.44 | 0.89 | 0.2 | 0 | 0 | 0 | 0.17 | 0 | 0 | 0 | 0 |
|  | **U2** | 1.53 | 1.14 | 0.93 | 0 | 0 | 0 | 0 | 0.12 | 0 | 0 | 0 | 0 |
| **11** | **B1** | 0.96 | 0.91 | 0.89 | 0.09 | 0 | 0 | 0 | 0 | 0 | 0 | 0 | 0 |
|  | **S1** | 0.96 | 0.87 | 0.93 | 0.09 | 0 | 0 | 0 | 0 | 0 | 0 | 0 | 0 |
|  | **S2** | 1.17 | 1.03 | 0.91 | 0.12 | 0 | 0 | 0 | 0 | 0 | 0 | 0 | 0 |
|  | **U1** | 1.16 | 1.46 | 1.04 | 0 | 0 | 0 | 0 | 0 | 0 | 0 | 0 | 0 |
|  | **U2** | 0.95 | 1.15 | 1 | 0.09 | 0 | 0 | 0 | 0 | 0 | 0 | 0 | 0 |

**Supplementary table 2**

| **pt ID** | | **D20S102** | **D20S149** | **RH64136** | **D20S459** | **D20S94** | **D20S171** |
| --- | --- | --- | --- | --- | --- | --- | --- |
| **1** | **B** | 182/184 | 279/295 | 220/222 | 228 | 138/144 | 133/139 |
|  | **S** | 182/184 | 279/295 | 220/222 | 228 | 138/144 | 133/139 |
|  | **U** | 182/184 | 279/295 | 220/222 | 228 | 138/144 | 133/139 |
| **2** | **B** | 180/184 | 273/309 | 214/220 | 228 | 138/144 | 125/133 |
|  | **S** | 180/184 | 273/309 | 214/220 | 228 | 138/144 | 125/133 |
|  | **father** | 180/182 | 273/291 | 220/222 | 228 | 138/144 | 125/133 |
|  | **mother** | 182/184 | 299/309 | 214/220 | 228 | 138/144 | 125/133 |
| **3** | **B** | 182/184 | 279/287 | 226/232 | 228 | 144 | 133/141 |
|  | **S** | 182/184 | 279/287 | 226/232 | 228 | 144 | 133/141 |
| **4** | **B** | 182/186 | 283/295 | 218/224 | 226/228 | 138/144 | 135 |
| **5** | **B** | 182 | 287/307 | 218/226 | 228 | 144 | 129/131 |
|  | **S** | 182 | 287/307 | 218/226 | 228 | 144 | 129/131 |
|  | **U** | 182 | 287/307 | 218/226 | 228 | 144 | 129/131 |
| **6** | **B** | 180/184 | 283/287 | 216/218 | 232 | 138/144 | 133/137 |
|  | **S** | 180/184 | 283/287 | 216/218 | 232 | 138/144 | 133/137 |
| **7** | **B** | 180/184 | 281/293 | 220/226 | 228 | 144 | 125/135 |
|  | **S** | 180/184 | 281/293 | 220/226 | 228 | 144 | 125/135 |
|  | **U** | 180/184 | 281/293 | 220/226 | 228 | 144 | 125/135 |
| **8** | **B** | 184 | 277/297 | 218 | 234 | 146 | 139 |
|  | **S** | 184 | 277/297 | 218 | 234 | 146 | 139 |
| **9** | **B** | 184/186 | 287 | 222/228 | 228 | 138/144 | 131/133 |
|  | **S** | 184/186 | 287 | 222/228 | 228 | 138/144 | 131/133 |
|  | **U** | 184/186 | 287 | 222/228 | 228 | 138/144 | 131/133 |
| **10** | **B** | 180/184 | 287/311 | 220/226 | 238 | 146/152 | 131/141 |
|  | **S** | 180/184 | 287/311 | 220/226 | 238 | 146/152 | 131/141 |
|  | **U** | 180/184 | 287/311 | 220/226 | 238 | 146/152 | 131/141 |
| **11** | **B** | 180/182 | 287/299 | 204/220 | 238/248 | 144 | 131/141 |
|  | **S** | 180/182 | 287/299 | 204/220 | 238/248 | 144 | 131/141 |
|  | **U** | 180/182 | 287/299 | 204/220 | 238/248 | 144 | 131/141 |
| Legend: B (blood), S (saliva), U (urine).  Numbers in columns under markers’ names represent the observed amplicon lenght, that changes according with the number of repeats of the VNTR. The presence of only one number means that the sample was homozygous for such genetic marker. | | | | | | | |

**Supplementary Figure 1.** Methylation-specific probes location. GR37/hg19.


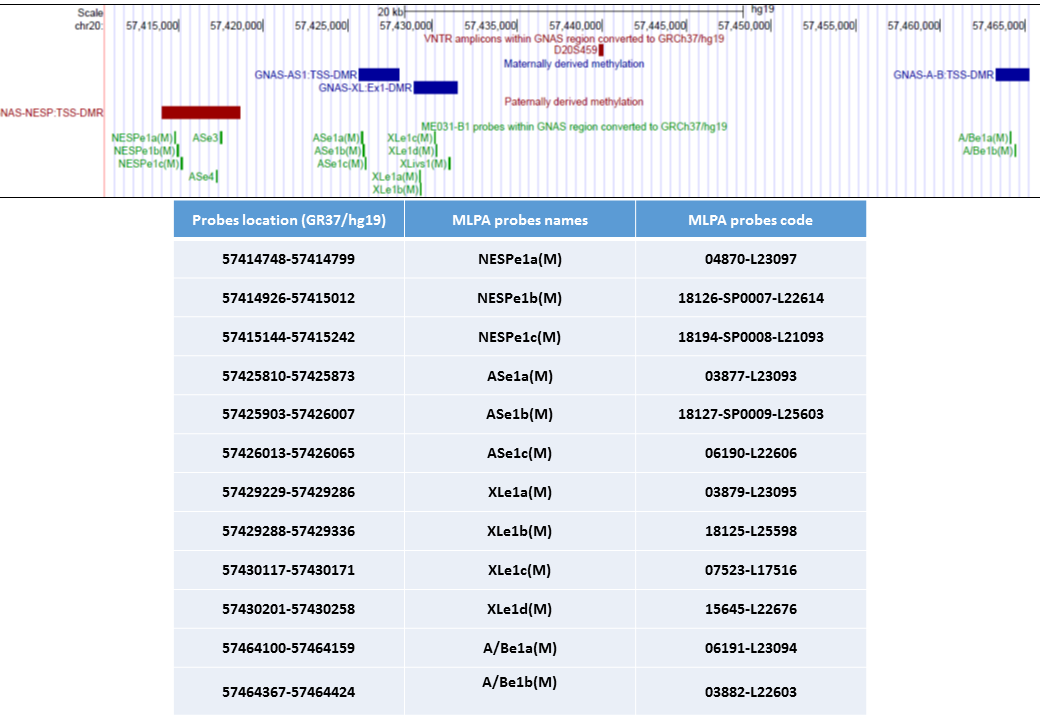

Supplement: Additional file 1: Figure S1. — Methylation-specific probes location, GR37/hg19. Table S1. Raw methylation ratios. Table S2. Variable number tandem repeats (VNTRs) in the 20q region analysed to exclude uniparental disomy (UPD). (DOCX 378 kb) [file 13148_2018_449_MOESM1_ESM.docx]
